# Differentiation of Bone Marrow Mesenchymal Stem Cells into Neural Lineage Cells Induced by bFGF-Chitosan Controlled Release System

**DOI:** 10.1155/2019/5086297

**Published:** 2019-03-27

**Authors:** Manli Li, Wen Zhao, Yudan Gao, Peng Hao, Junkui Shang, Hongmei Duan, Zhaoyang Yang, Xiaoguang Li

**Affiliations:** ^1^Beijing Key Laboratory for Biomaterials and Neural Regeneration, School of Biological Science and Medical Engineering, Beihang University, 100083 Beijing, China; ^2^Beijing International Cooperation Bases for Science and Technology on Biomaterials and Neural Regeneration, Beijing Advanced Innovation Center for Big Data-Based Precision Medicine, Beihang University, 100083 Beijing, China; ^3^Beijing Advanced Innovation Center for Biomedical Engineering, Beihang University, 100083 Beijing, China; ^4^Department of Neurobiology, School of Basic Medical Sciences, Capital Medical University, 100069 Beijing, China

## Abstract

Bone marrow mesenchymal stem cells undergo differentiation to different lineages with different efficiencies when induced by different factors. We added a bFGF-chitosan controlled release system (bFGF-CCRS) as an inducer into conditioned medium to facilitate the oriented differentiation of BMSCs into neural lineage cells (eventually mature neurons); furthermore, we synchronized BMSCs to the G0/G1 phase via serum starvation to observe the effect of the inducer on the differentiation direction and efficiency. The nonsynchronized group, chitosan alone (not loaded with bFGF) group, soluble bFGF group, and conditioned medium group served as controls, and we observed the dynamic process of differentiation of BMSCs into neural lineage cells at different time points after the beginning of coculture. We analyzed the binding patterns of bFGF and chitosan and assayed the expression differences of key factors (FGFR1, ERK, and c-fos) and molecular switches (BTG2) that regulate the transformation from cell proliferation to differentiation. We also investigated the potential molecular mechanism of BMSC differentiation into neural lineage cells at a high percentage when induced by bFGF-CCRS.

## 1. Introduction

Many neurodegenerative diseases have been shown to be associated with the degeneration of specific types of neurons accompanied by functional loss. Embryonic stem cells and neural stem/progenitor cells are usually considered candidate cells for cell transplantation to treat these diseases in clinical trials [1, 2], but there are some limitations in the clinical setting. For instance, immunological rejection, insufficient tissue supply, treatment scale, and ethical issues are common limitations. The contamination of glial cells during the neural induction process should not be neglected as well.

Bone marrow mesenchymal stem cells (BMSCs) originating from bone marrow are regarded as the best candidates for cell replacement. They have advantages including ease of isolation, strong proliferation capacity, and immunological naivety, and there are no ethical issues concerning their use 6. Under specific conditions, BMSCs can differentiate into other cell types, including osteoblasts, adipose cells, and chondrocytes [3]. According to some in vitro experimental results, when BMSCs were induced to differentiate into neurons, they also generated glial cells [4, 5]. When BMSCs were exposed to an environment harboring FGF-2, FGF-8, brain-derived neurotrophic factors (BDNF), or some special substrates, respectively, they could be induced to differentiate into neurons [6]. Overall, BMSCs may serve as good candidates for cell replacement in the repair and regeneration of neural tissue.

In fact, BMSCs cannot always differentiate into neurons at satisfactory efficiencies and yields, and experimental results often fluctuated by batch. Additionally, because of very short half-life under physiological conditions [7], it is difficult for soluble neurotrophic factors to reside at the diseased/injured site and function effectively. To overcome these shortcomings, we tentatively combined the neurotrophic factor bFGF with a degradable chitosan scaffold to prolong its half-life in a physiological environment. Chitosan has good histocompatibility and is widely used in tissue engineering. Next, we cocultured this bioactive scaffold with BMSCs from rat to improve the survival and adhesion of BMSCs as well as their oriented differentiation into neurons. This interdisciplinary approach based on tissue engineering may shed light on tissue repair and functional recovery [8]. Functioning as a physical scaffold, the chitosan scaffold may facilitate cell adhesion, growth, proliferation, and further differentiation [9]. In addition, this bioactive scaffold can also serve as a controllable release system to control bFGF release for up to several weeks, which further facilitates the proliferation and differentiation of BMSCs and ultimately improves their differentiation into neurons. As previously reported, embryonic stem cells and neural precursors have been synchronized to the G0/G1 phase through serum starvation, which enabled the improved differentiation of neural precursor cells into neurons [10, 11]. In this study, we used serum starvation to achieve cell cycle synchronization of BMSCs to the G0/G1 phase and cocultured synchronized BMSCs with a bioactive bFGF-chitosan scaffold to observe the impact of cell cycle synchronization on BMSC differentiation into neurons and explore the underlying mechanism. This approach may provide new insights into the clinical treatment of nervous system diseases and injuries.

## 2. Materials and Methods

### 2.1. Preparation of bFGF-Chitosan Scaffold

Under sterile conditions, 10 mg of 85% deacetylated chitosan particles (Sigma, St.Louis, USA) was dissolved in 10 ml deionized water, allowed to swell for 6 h, and centrifuged. Then the supernatant was discarded. The swollen chitosan particles were frozen at -20°C for 24 h and then placed at 4°C for 10 h. 20 ng bFGF (Yisheng, Zhuhai, China) was dissolved in 1 ml cold deionized water and then added to the abovementioned 4°C chitosan particles. After stirring at 4°C for 6 h, the mixture was vacuum cooled and dried. The dried chitosan particles were added to type I collagen solution at 4°C, stirred for 30 min, centrifuged, collected, and stored at 4°C for use.

### 2.2. FTIR-ATR Characterization

FTIR-ATR (attenuated total reflection Fourier transform infrared spectroscopy) was used to study the binding patterns of bFGF and chitosan. For FTIR, 2.5 mg freeze-dried bFGF powder, chitosan alone (not combined with bFGF), and bFGF-chitosan scaffolds were ground with a mortar and pestle, respectively, mixed with 500 mg KBr, and compressed with 10 tons (diameter of compaction = 13 mm) under vacuum for 1 min before analysis. The FTIR spectra were acquired between 4000 and 600 cm^−1^.

### 2.3. TGA

To test the thermodynamic stabilities of bFGF, chitosan, and bFGF-chitosan scaffolds, we conducted TGA (thermogravimetric analysis) at temperatures ranging from 25°C (room temperature) to 800°C and recorded the real-time TGA and DTG (differential thermogravimetric) values of these samples.

### 2.4. Extraction and Purification of BMSCs

Under sterile conditions, Equithesin was injected into 7-day-old Wistar rats (0.3 ml/100 g body weight). After anesthesia overdose death, the thigh and shin bones of these animals were removed by an operation and washed with 0.01 M PBS to expose the two cutting ends, and the marrow contents were expelled using a syringe in a culture dish containing 10% FBS (fetal bovine serum, Gibco, USA) supplemented DMEM (Thermo Fisher Scientific, USA) culture medium, where BMSCs were rinsed and dissociated into single cells as much as possible. These cells were cultured in a 5% CO_2_ humidified incubator at 37°C. Twenty-four hours later, nonadherent cells were removed by a full medium change. When cell confluency reached 80-90%, 0.25% trypsin (Gibco, USA) was used to digest BMSCs for 2 min. Next, the cell suspension was centrifuged at 1000 rpm (revolutions per min) for 5 min, and the supernatant was discarded. After 2 ml trypsin was added, the sedimented cells were dissociated into single cells. Then, the cell suspension was evenly divided and placed into two culture dishes containing 10 ml DMEM culture medium with 10% FBS and cultured in a 5% CO_2_ humidified incubator at 37°C until passage 3 for future use. [12–14]

### 2.5. Identification of BMSCs by Flow Cytometry

P3 BMSCs were seeded at a cell density of 10^5^ cells/ml in a 6-well plate coated with 25 *μ*mol/L poly-L-lysine (Sigma-Aldrich, USA). When cell confluency reached 80-90%, these BMSCs were rinsed with 0.01 M PBS twice and digested with 2 ml of 0.25% trypsin for 2 min. Finally, the same volume of culture medium was added to quench the digestion. The adherent cells were removed from the surface of the wells, collected into an EP tube, and centrifuged at 1000 rpm for 10 min. The cells were washed with 0.01 M PBS, and fluorescent-labeled anti-CD90-FITC (Abcam, USA), anti-CD105-FITC (Abcam, USA), anti-CD73-FITC (Abcam, USA), anti-CD45-Cy5 (Abcam, USA), anti-CD34-Cy5 (Abcam, USA), and anti-CD31-Cy5 (Abcam, USA) antibodies were added, incubated on ice for 40 min, and assayed on a flow cytometer (FACS Calibur, Becton Dickinson).

### 2.6. BrdU Incorporation

When cells confluency reached 80-90%, cells were incubated in a 37°C incubator for 45 min with 10 ml of fresh medium containing 0.2% BrdU (Sigma-Aldrich, USA). Then, the cells were rinsed with 0.01 M PBS twice and fixed in a 4°C environment with 4°C 4% PFA (paraformaldehyde) (Sigma-Aldrich, USA) for 40 min. Next, the cells were denatured with 2 M hydrochloric acid for 15 min and then neutralized with 0.1 M sodium tetraborate for 15 min. After the denatured cells were blocked with 10% serum for 30 min, they were incubated with BrdU antibody (mouse, CST, 1:200) for 2 hours. Next, excess primary antibody was washed off, and fluorescent-labeled secondary antibody was added for immunofluorescence. Cells were observed with a fluorescence microscope (BX51, Olympus).

### 2.7. Western Blot

At 1, 3, 7, 10, and 14 days after coculture, cells were washed three times with PBS and then lysed with RIPA Lysis Buffer (Beyotime Institute of Biotechnology, Jiangsu, China) supplemented with 1 mM PMSF (Roche Molecular Biochemicals, Mannheim, Germany). BCA (Beyotime Institute of Biotechnology, Jiangsu, China) protein assay kit was used to measure protein concentration of each group. Aliquots of total protein were separated by 8% sodium dodecyl sulfate polyacrylamide gel electrophoresis (SDS-PAGE) and then transferred to polyvinylidene fluoride (PVDF) membranes (Bio-Rad Laboratories, Hercules, USA). Membranes were blocked in 5% nonfat milk for 1 h at room temperature and subsequently incubated with primary antibodies: rabbit anti-FGFR1 (Abcam, Cambridge, U.K.) (1: 2000 dilution); rabbit anti-c-fos (Abcam, Cambridge, U.K.) (1: 1000 dilution); and rabbit anti-ERK (Abcam Cambridge, U.K.) (1: 2000 dilution) overnight at 4°C, respectively. After three washes, membranes were incubated with horseradish peroxidase-(HRP-) conjugated goat anti-rabbit IgG for 1 h, washed again with TBST, and subsequently visualized using West-Pico ECL kit (Pierce, Rockford, USA) and GeneSys (Gene, H.K). Image J was used to analyze the densitometries in different groups.

### 2.8. Cell Cycle Synchronization of BMSCs

P3 BMSCs were seeded at a cell density of 10^5^ cells/ml in a 6-well plate coated with poly-L-lysine. After cells adhered to the surface, the cells were processed via serum starvation as follows. The culture medium was changed to DMEM containing 10, 5, 1, 0.5, 0.1, and 0% FBS and cultured in an incubator for 12, 24, 36, 48, and 72 hours, respectively. Next, the cells were rinsed with 0.1 M PBS three times. The adherent cells were digested with 0.25% trypsin into a cell suspension and collected into an EP tube, where the cells were fixed with 70% ethanol for 2 hours. After PI (propidium iodide) (Sigma-Aldrich, USA) staining for 10 s, a flow cytometer (Beckman, EPICS XL coulter) was used to detect the percentage of G0/G1, G2/M, and S cells for every 10^4^ cells. The experiment was repeated five times for each group.

### 2.9. Calcein AM/PI Staining

Serum-starved cells were digested with trypsin-EDTA, and the cells were collected in centrifuge tubes and centrifuged at 1000 rpm for 3 min. The supernatant was removed, and the cell suspension was adjusted to 10^5^-10^6^ cells/ml with PBS and then mixed gently. This process was repeated several times to completely remove the medium. Then, 200 *μ*l of the cell suspension was incubated at 37°C for 15 min after adding 100 *μ*l of staining solution (calcein AM/PI) (Sigma-Aldrich, USA). Yellow-green living cells and red dead cells were observed at an excitation wavelength of 490 nm and 545 nm.

### 2.10. Neural Lineage Induction

Before the initiation of neural lineage induction, P3 BMSCs were planted in a six-well plate at a cell density of 10^5^ cells/ml and were cultured in specific medium for neural lineage cells as follows: DMEM/F12 (Gibco, USA) with B27 (Gibco, USA), 20 *μ*g/ml bFGF (Sigma-Aldrich, USA), and 20 *μ*g/ml EGF (Sigma-Aldrich, USA). Next, the BMSCs cells were cocultured with either (1) soluble bFGF (20 ng/ml bFGF was added once at the beginning of the coculture); (2) chitosan scaffold alone (10 mg/ml chitosan scaffold without bFGF was added once at the beginning of the coculture); or (3) bFGF-chitosan scaffold (10 mg/ml bFGF-scaffold, i.e., loaded with 20 ng/ml bFGF, was added once at the beginning of the coculture). Fifty percent of the medium in each well was exchanged every 3 days. Corresponding indexes were detected at specific time points.

### 2.11. Immunofluorescent Staining

At 3, 7, 10, 14, and 18 days after coculture, cells were washed with 0.01 M PBS three times and fixed with 4°C 4% PFA in a 4°C environment for 40 min. Next, after washing off PFA with 0.01 M PBS, the cells were incubated with 0.3% PBST for 5 min and blocked with 10% NGS (normal goat serum) at 37°C for 30 min. Then, the cells were incubated with primary antibodies at 37°C for 2 hours. When the excess primary antibodies were washed off with PBS three times, the cells were incubated with secondary antibodies at 37°C for 50 min (all antibodies and dilutions are listed in Table 1). Then the samples were washed with PBS three times. Finally, nuclei were stained with DAPI (Sigma-Aldrich, USA). The stained cells were examined with a fluorescence microscope equipped with a digital camera (TCS SP8 laser confocal microscope, Leica).

### 2.12. QRT-PCR (Quantitative Reverse Transcription-PCR)

A TRIzol extraction method [15] was used to extract mRNA from each group to synthesize first-strand cDNA (SuperScript™ III First-Strand Synthesis System for RT-PCR, Invitrogen, Cat. No: 18080-051). Using Primer 6, primers were designed using cDNA as the template. All primer information is listed in Table 2. Relative mRNA levels were quantified by a real-time PCR assay using an Applied Biosystems 7900 Fast real-time PCR system.

### 2.13. Data Presentation and Statistical Analysis

All data are shown as the mean±SEM unless stated otherwise. One-way ANOVA and Bonferroni analysis (multiple comparisons for three groups) were used for statistical analysis.* P*<0.05 was considered to indicate a statistically significant difference.

All experimental procedures were approved by and performed in accordance with the standards of the Experimental Animal Center of Capital Medical University and the Beijing Experimental Animal Association.

## 3. Results

### 3.1. Characterization of Thermodynamic Stability by FTIR-ATR

**Figure 1:**
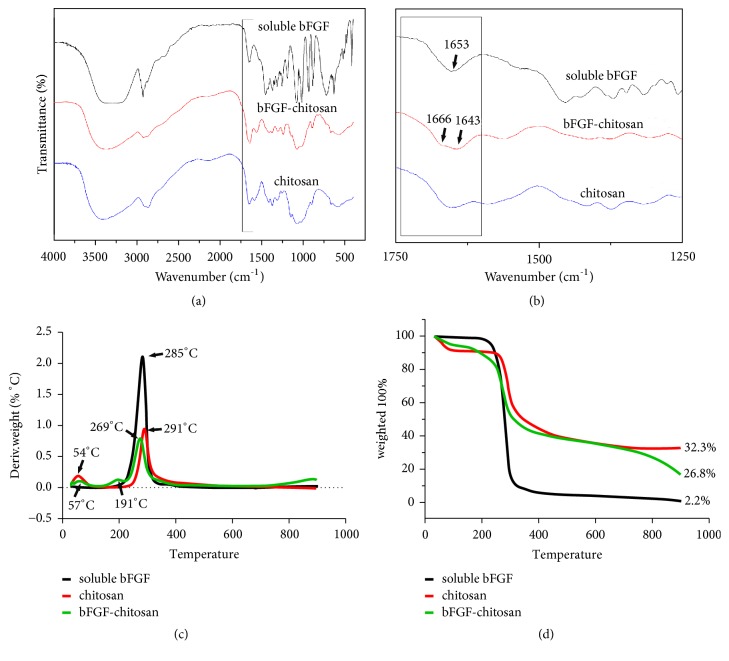
FTIR-ATR characterization and thermogravimetric analysis. (a) FTIR transmission spectra of soluble bFGF (sbFGF), the bFGF-chitosan controlled release scaffold (bFGF-chitosan), and chitosan alone (chitosan) to reveal the combined effect of bFGF and chitosan; (b) amplification of image (a) at 1500 cm^−1^; and (c-d) TGA and DTG of soluble bFGF, the bFGF-chitosan controlled release scaffold, and chitosan alone to reveal their thermal stability. The arrow refers to the difference between the three groups.

To investigate the binding pattern of chitosan and bFGF, we conducted FTIR analysis on soluble bFGF, chitosan alone, and bFGF-chitosan controlled release scaffolds. Compared with the soluble bFGF and chitosan alone groups, the results suggest that, in the bFGF-chitosan controlled release scaffold group, the position of the absorption peak that corresponded to the stretching vibration of double-bond C=O changed as follows: the stretching vibration peak of C=O split into two peaks, with peak values of 1643 and 1666 cm^−1^ (Figures 1(a) and 1(b)), indicating that both bFGF and chitosan may have formed hydrogen bonds between C=O groups.

To further investigate the thermal stability of the bFGF-chitosan controlled release scaffold, we analyzed the TGA curves of these three materials and obtained the DTG curves. These curves suggest that as the temperature increased from room temperature to 800°C, bFGF experienced one weight loss, which peaked at a decomposition temperature of 285°C. Furthermore, chitosan experienced two weight losses, which peaked at decomposition temperatures of 57°C and 291°C because of water loss and decomposition of the chitosan scaffold, respectively. Finally, the bFGF-chitosan controlled release scaffold also experienced two weight losses, which peaked at decomposition temperatures of 54°C and 269°C. Compared with soluble bFGF and chitosan alone, both decomposition temperatures for the bFGF-chitosan controlled release scaffold shifted towards the left, thus lowering its thermal stability (Figures 1(c) and 1(d)). The TGA, DTG, and FTIR results for the bFGF-chitosan controlled release scaffold suggested an interaction between bFGF and chitosan in which the double-bond C=O participated. This interaction, likely due to hydrogen bonding, might slightly reduce the thermal stability of the bFGF-chitosan controlled release scaffold.

### 3.2. Primary Culture and Identification of BMSCs

**Figure 2:**
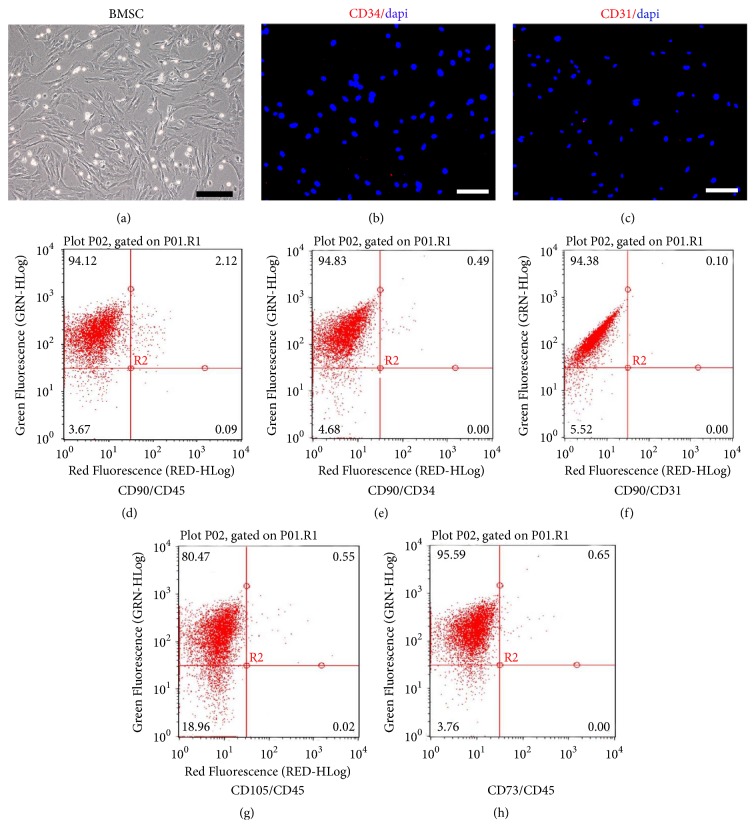
Morphological observation of P3 BMSCs and characterization by flow cytometry and immunofluorescence. (a) Morphological observation of P3 BMSCs. The cells were fibroblast-like shaped and possessed a screw-like alignment. (b-c) Immunofluorescent staining of CD34 and CD31, respectively. (d) Expression of CD90 and CD45, respectively, in P3 BMSCs by flow cytometry. Upper-left: CD90^+^/CD45^−^ cells comprised approximately 94.12% of the total cells. (e) Expression of CD90 and CD34 in P3 BMSCs by flow cytometry. Upper-left: CD90^+^/CD34^−^ cells comprised approximately 94.83% of the total cells. (f) Expression of CD90 and CD31 in P3 BMSCs by flow cytometry. Upper-left: CD90^+^/CD31^−^ cells comprised approximately 94.38% of the total cells. (g) Expression of CD105 and CD45 in P3 BMSCs by flow cytometry. Upper-left: CD105^+^/CD45^−^ cells comprised approximately 80.47% of the total cells. (h) Expression of CD73 and CD45 in P3 BMSCs by flow cytometry. Upper-left: CD73^+^/CD45^−^ cells comprised approximately 95.59% of the total cells.

The proportion of BMSCs is extremely low in the bone marrow cell population. As previously reported, multiple types of cells influence the differentiation direction. Therefore, we detected the cell surface markers CD31 and CD34 by immunofluorescence and CD90, CD45, CD105, and CD73 by flow cytometry. The results suggested that over 95% of cells were CD90^+^ and CD45^−^, CD73^+^ and CD45^−^, and CD105^+^ and CD45^−^ and almost all cells were CD31^−^ and CD34^−^. Considering that these cells were adherent to the wall of the plastic well with a fibroblast-like shape, we speculated that over 95% of the cells we extracted and purified were BMSCs (Figure 2).

### 3.3. BMSCs Were Induced Morphologically into Neurosphere-Like Cells

**Figure 3:**
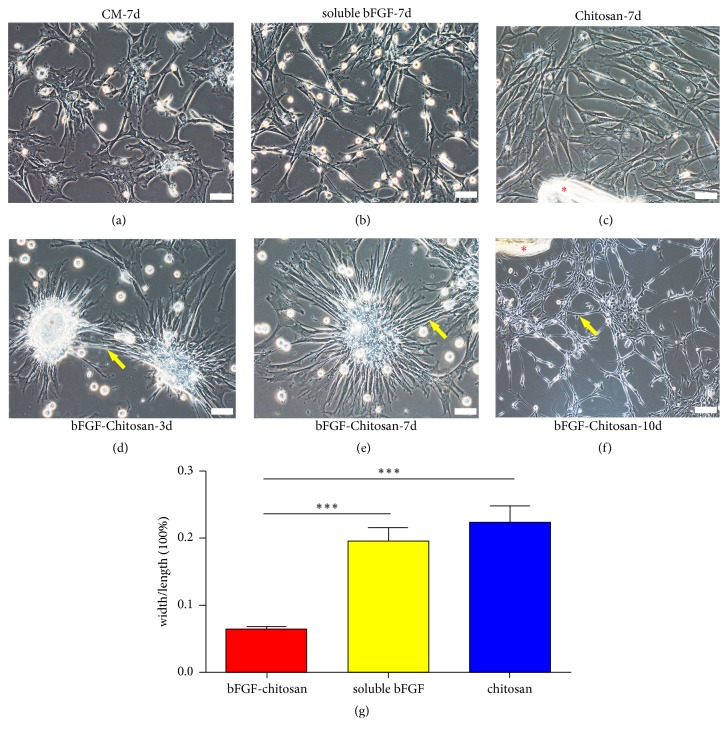
Morphological observation of BMSCs and differentiated cells with phase contrast microscopy. (a) BMSC morphology after 7 days of coculture in conditioned medium (CM). The cells did not show noticeable morphological changes compared with P3 BMSCs and retained a fibroblast-like shape. (b) BMSC morphology after 7 days of coculture with 20 ng soluble bFGF. (c) BMSC morphology after 7 days of coculture with chitosan alone. (d-f) Cell morphology after 3, 7, and 10 days of coculture with bFGF-chitosan: (d) after 3 days of coculture, the cells began to aggregate into neurosphere-like structures; (e) after 7 days of coculture, processes increasingly grew out of the neurosphere-like structures and formed contacts with adjacent neurosphere-like cells; and (f) after 10 days of coculture, the cell morphology substantially changed, and a network formed among cells. (g) Quantitative analyses of the ratios of process length to width. Data represent the means ± SE; bFGF-chitosan vs. bFGF, bFGF-chitosan vs. chitosan *∗∗∗ P* <0.001. *∗* is the undegraded chitosan scaffold, scale bar for (a)-(e) is 50 *μ*m and for (f) is 100 *μ*m.

To observe and evaluate the potential differentiation capacity of BMSCs from rat into neural lineage cells induced by the bFGF-chitosan scaffold, we cocultured P3 BMSCs with conditioned medium (DMEM/F12 with B27, 20 ng/ml bFGF, and 20 ng/ml EGF), soluble bFGF, chitosan alone, or the bFGF-chitosan scaffold, respectively. At 7 days after coculture, there was no significant morphological change observed in the conditioned medium group and chitosan alone group. These BMSCs retained a fibroblast-like shape (Figures 3(a) and 3(c)). BMSCs showed some small and short processes (Figure 3(b)) in the soluble bFGF group. However, in the bFGF-chitosan scaffold group, the cells displayed a neurosphere-like morphology at 3 days after coculture (Figure 3(d)). After 7 days of coculture, the neurosphere-like cells had more processes (Figure 3(e)) and 10 days after coculture, the processes increased continuously and formed a network between each other (Figure 3(f)). Considering the distinct morphology of neural cells, i.e., less cytoplasm and longer processes [16], we arbitrarily selected 10 cells from each group and calculated the ratio of cell width to length after 7 days of coculture to analyze cell morphology. The results suggested that there were significantly smaller cell width to length ratios in the bFGF-chitosan scaffold group than those in the other two groups (Figure 3), indicating that the bFGF-chitosan scaffold enabled efficient differentiation of BMSCs into neurosphere-like cells.

### 3.4. BMSCs Were Induced to Differentiate into Neurons via the Transdifferentiation of Nestin-Positive Neural Stem Cells

**Figure 4:**
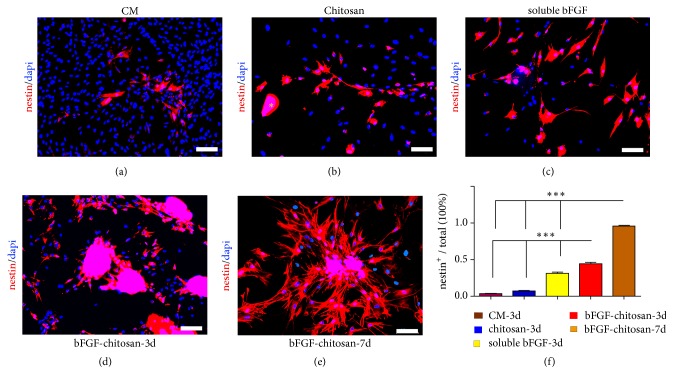
BMSCs were induced to express nestin. (a) Nestin expression after 7 days of coculture in the conditioned medium. (b) Nestin expression after 7 days of coculture with chitosan that was added once at the beginning of the coculture. Yellow *∗* indicates the undegraded chitosan scaffold. (c) Nestin expression after 7 days of coculture with soluble bFGF (20 ng/ml) that was added once at the beginning of the coculture. (d) Nestin^+^ expression after 3 days of coculture with bFGF-chitosan that was added once at the beginning of the coculture. (e) Nestin^+^ expression after 7 days of coculture with bFGF-chitosan that was added once at the beginning of the coculture. The results suggest that, along with coculture time extension, the nestin expression percentage was substantially increased in the bFGF-chitosan group. (f) Quantitative analyses of nestin^+^ cell percentage in each group. Data represent the means ± SE. bFGF-chitosan-3d vs. the other three groups and bFGF-chitosan-7d vs. the other three groups *∗∗∗P* <0.001. Scale bar: 100 *μ*m.

After 3 days of coculture, cells were immunostained with nestin, the neural stem cell marker. The results suggested that there were only a few nestin^+^ cells in the chitosan alone, conditioned medium, and soluble bFGF groups, while there were a large number of nestin^+^ cells in the bFGF-CCRS group (95.8±0.96%) (Figure 4).

**Figure 5:**
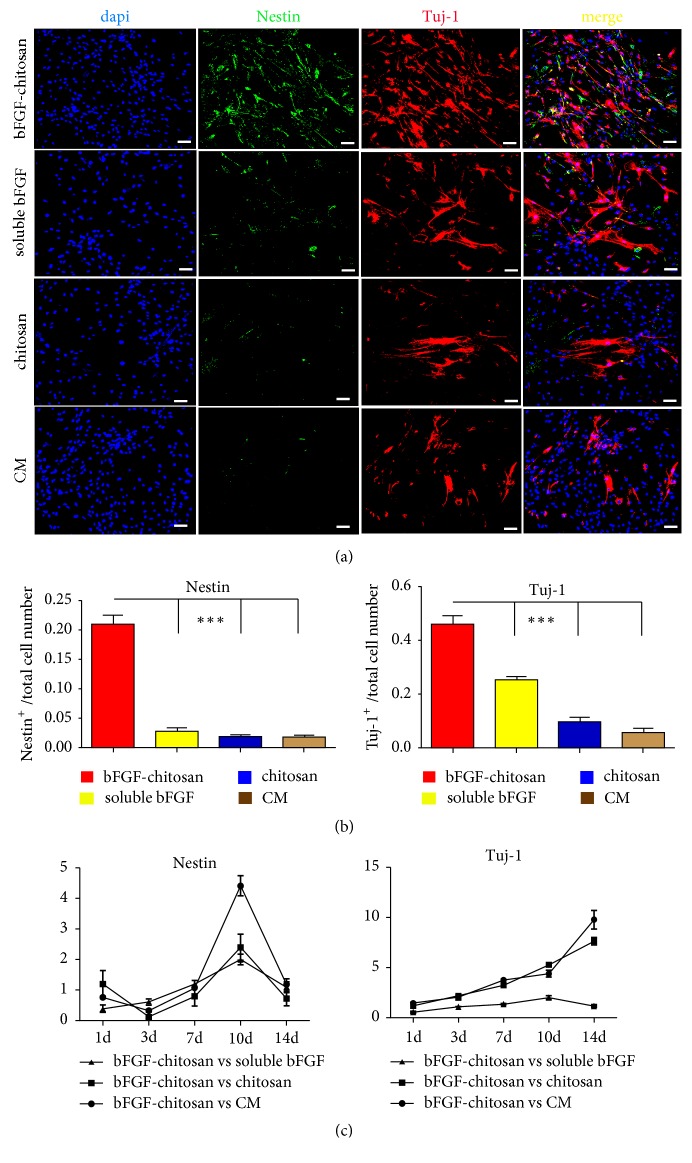
Under different coculture conditions, BMSCs were induced to differentiate into Tuj-1^+^ cells after 14 days of coculture. (a) bFGF-chitosan could significantly facilitate the differentiation of BMSCs into Tuj-1^+^ cells. (b) Quantitative analysis of Tuj-1^+^ cell numbers under different coculture conditions. (c) Qrt-PCR analysis mRNA expression levels of nestin and Tuj-1 at different time points. Data represent the means ± SE. bFGF-chitosan vs. bFGF, bFGF-chitosan vs. conditioned medium *∗∗∗P* <0.001. Scale bar: 100 *μ*m.

**Figure 6:**
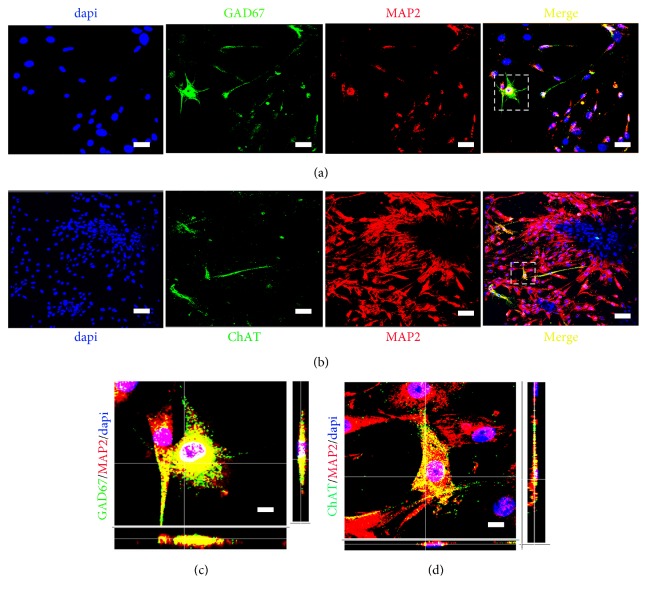
bFGF-chitosan induced BMSCs to differentiate into GAD67^+^ and ChAT^+^ cells. (a) After 18 days of coculture, bFGF-chitosan induced BMSCs to differentiate into MAP_2_^+^/GAD67^+^ cells. (b) After 18 days of coculture, bFGF-chitosan induced BMSCs to differentiate into MAP_2_^+^/ChAT^+^ cells. (c) Amplification of image (a). (d) Amplification of image (b). Scale bar: (a)-(b) 80 *μ*m; (c)-(d) 10 *μ*m.

In the bFGF-CCRS group, on day 14 of coculture, a large number of Tuj1^+^ immature neurons were observed, significantly more than those in the other three groups (*P*=0.000035,* P*=0.000078, and* P*=0.000057). Furthermore, nestin and Tuj1 double-positive cells were also observed (Figure 5) and on day 18, ChAT^+^ and GAD67^+^ cells were observed (Figure 6). Collectively, along with the coculture progression, the bFGF-chitosan scaffold increased the transdifferentiation percentage of BMSCs into nestin^+^ neural stem cells, indicating that it could improve the differentiation capacity of BMSCs into neurons and facilitate neuronal maturation into cholinergic and GABAergic neurons.

### 3.5. Upregulation of the MAPK Signaling Pathway by bFGF-CCRS

**Figure 7:**
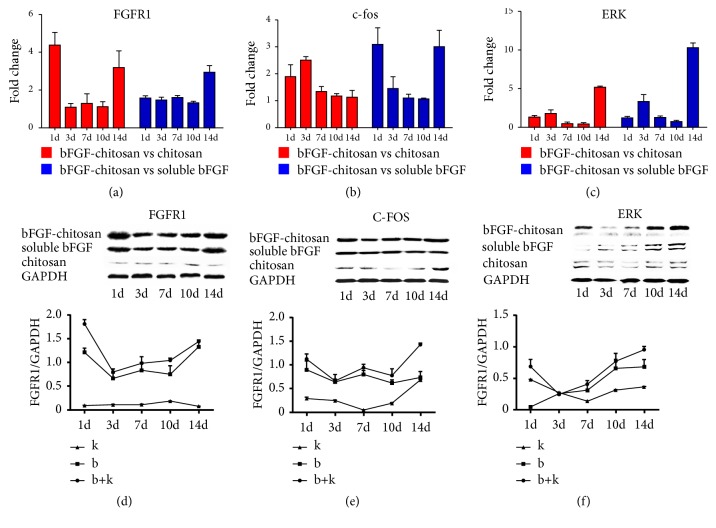
The key factors (c-fos and ERK) in the MAPK signaling pathway were effected by FGFR1 in different coculture system. (a) Expression of the bFGF receptor FGFR1 at different time points. bFGF-chitosan vs. soluble bFGF or bFGF-chitosan vs. chitosan. (b) Expression of the transcription factor c-fos in the MAPK signaling pathway. bFGF-chitosan vs. soluble bFGF or bFGF-chitosan vs. chitosan. (c) Expression of the transcription factor ERK in the MAPK signaling pathway. bFGF-chitosan vs. soluble bFGF or bFGF-chitosan vs. chitosan. (d-f) WB and densitometry are used to measure expression of protein of the bFGF receptor FGFR1 and key factors (c-fos and ERK) at different time points. bFGF-chitosan vs. soluble bFGF or bFGF-chitosan vs. chitosan. Data represent the means ± SE.

To determine the cause of the improved differentiation percentage of BMSCs into neural cells by the bFGF-chitosan scaffold, the expression of key genes was studied in the bFGF-related signaling pathway. It has been suggested that bFGF functions mainly by combining with the cell surface receptor FGFR1 to activate relevant downstream proteins and regulate the MAPK signaling pathway [17]. The MAPK signaling pathway, one of the important signal transduction systems, was involved in multiple biological and pathological processes, including cell growth, development, division, and differentiation, with ERK and c-fos as key proteins in the signaling pathway [18]. Our qRT-PCR results suggested that the expression levels of FGFR1, ERK, and c-fos were significantly upregulated in the bFGF-CCRS group compared with those in the soluble bFGF, chitosan alone, and conditioned medium groups (Figures 7(a)–7(c)), and our western blot results also suggested the expression levels of these protein were significantly upregulated (Figures 7(d)–7(f)), indicating that the bFGF-chitosan scaffold might increase the expression level of FGFR1 to activate the downstream MAPK signaling pathway and consequently improve the differentiation percentage of BMSCs into neural cells.

### 3.6. Cell Cycle Synchronization Further Improved the Synergistic Effects of the MAPK Signaling Pathway

**Figure 8:**
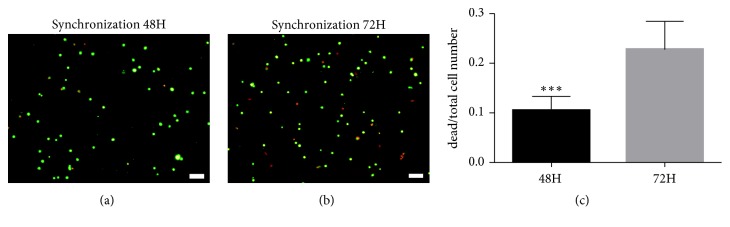
Cell viability was measured at 48 or 72 hours after treatment with 1% serum. (a) Cell viability after treatment with 1% serum for 48 hours. (b) Cell viability after treatment with 1% serum for 72 hours. (c) Quantitative analysis of cell viability in different groups. Red represents dead cells. Green represents living cells. Scale bar: (a)-(b) 100 *μ*m.

Cells that exit a certain cell cycle and enter the G0 phase may alter the cell fate, from proliferation to differentiation [19, 20]. During brain development, when cells exit a cell cycle, they differentiate into specific neurons or glial cells [21]. To improve the differentiation capacity, we used serum starvation [22] to synchronize BMSCs to the G0/G1 phase, that is, to make more cells exit their respective cell cycles and differentiate in the G0/G1 phase, thus increasing their differentiation capacity. To determine the optimal conditions for cell cycle synchronization, we processed BMSCs with 5, 1, 0.5, 0.1, and 0% FBS for 12, 24, 36, 48, and 72 hours, respectively, analyzed cell percentages in different phases by flow cytometry, and compared them with nonsynchronized cells. The results suggested that, along with decreasing FBS and increasing serum starvation time, the percentage of G0/G1 cells increased to some degree. Under the conditions of 1% FBS and 48 hours, the synchronized cells reached the peak of G0/G1 cell percentage (Table 3). In addition, cell viability was examined at 48 and 72 hours, respectively, using calcein AM/PI; fewer dead cells were observed at 48 hours than at 72 hours. Therefore, we selected 1% FBS and 48 hours as the optimal synchronization condition (Figures 8(a)–8(c)).

**Figure 9:**
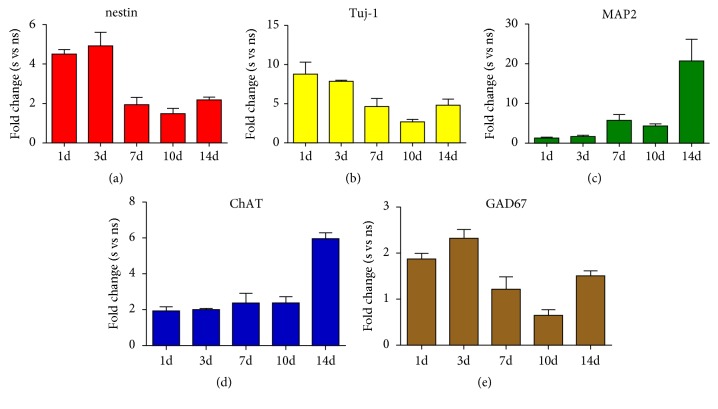
After cell cycle synchronization to the G0/G1 phase, the differentiation percentage of BMSCs into neural lineages was further increased under different coculture conditions than those nonsynchronized group. S indicates the synchronized group and NS indicates the nonsynchronized group. Data represent the means ± SE.

After cell cycle synchronization, the synchronized BMSCs were cocultured with conditioned medium, soluble bFGF, chitosan alone, or bFGF-CCRS, respectively, and their differentiation percentage into neural cells of each group was also detected. As shown by the qRT-PCR results, compared to the nonsynchronized control group, there were more nestin^+^, Tuj-1^+^, and MAP2^+^ cells in the bFGF-CCRS group after BMSCs were synchronized to the G0/G1 stage (Figures 9(a)–9(c)). The differentiation percentage of GAD67- and ChAT-positive cells also increased (Figures 9(d)-9(e)).

**Figure 10:**
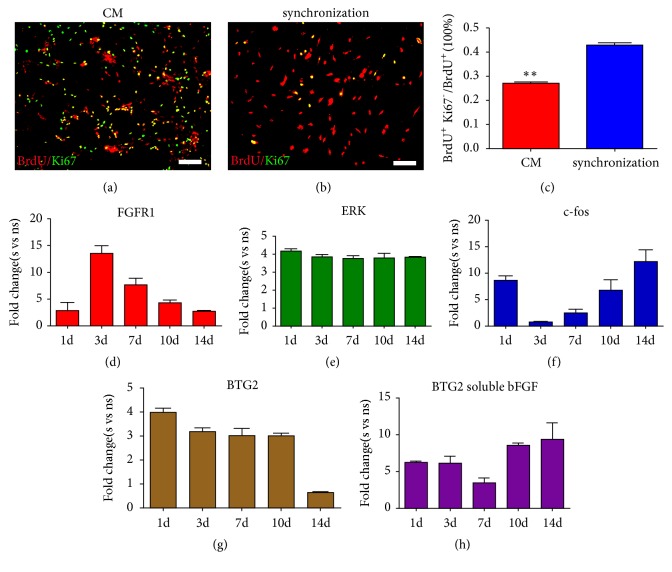
Cell cycle synchronization affects cell fate through MAPK signaling pathway. (a-b) BrdU and Ki-67 staining before and after cell cycle synchronization (BrdU^+^ is red and Ki67 is green). (c) Quantitative analysis of BMSC numbers at the G0 stage. Data represent the means ± SE; *∗∗*P <0.01. (d) Expression of the bFGF receptor FGFR1 at different time points for synchronized and nonsynchronized cells. S indicates the synchronized group and NS indicates the nonsynchronized group. (e) The expression of the c-fos transcription factor in the MAPK signaling pathway for synchronized and nonsynchronized cells. (f) The expression of the ERK transcription factor in the MAPK signaling pathway for synchronized and nonsynchronized cells. (g) The expression difference of the molecular switch BTG2, which regulates the transformation from cell proliferation to differentiation, before and after cell cycle synchronization. (h) The expression of BTG2 for the group of soluble bFGF and condition medium. Data represent the means ± SE.

We quantitatively analyzed the G0/G1 cell percentages after cell cycle synchronization and double-labeled the synchronized cells with BrdU and Ki-67. Because Ki-67 is expressed in all cell cycle stages except for the G0 phase, we used Ki-67^−^ and BrdU^+^ to designate G0/G1 cells [23]. The results suggested that cell cycle synchronization improved the percentage of G0/G1 cells and made more cells differentiate (Figures 10(a)–10(c)). Next, we tested the expression of the BTG2 molecular switch that controls the transformation from proliferation to differentiation and found that BTG2 expression was significantly improved compared with the nonsynchronized group at every time point (day 1* P*=0.001, day 3* P*=0.001, day 7* P*=0.004, day 10* P*=0.002, and day 14* P*=0.001). This also suggested that more cells had their fate changed from proliferation to differentiation, thus facilitating the differentiation percentage of BMSCs into neural lineages (Figures 10(f)–10(h)). We also investigated the expression levels of BTG in the soluble bFGF cells before and after synchronization, and no significant changes were found, indicating that the improvement of the differentiation ratio may be achieved by the bFGF-CCRS and synchronization. Additionally, we examined the expression of key factors in the MAPK signaling pathway and observed that the expression levels of key genes (ERK and C-fos) in the MAPK signaling pathway were increased in the synchronized group compared with those in the nonsynchronized group (day 1* P*=0.0006, day 3* P*=0.0001, day 7* P*=0.00003, day 10* P*=0.0005, and day 14* P*=0.0001) (Figure 10(d)).

## 4. Discussion

In previous studies, NSCs were used as the primary cell source to induce cell differentiation. However, because of their limited and dormant populations in adults, difficult acquisition, and secondary damage to the body, NSCs are considered not suitable for the treatment of some central nervous system (CNS) degenerative diseases and CNS traumatic injuries, which are caused by the loss of neural cells (especially neurons). Therefore, it is very advantageous to use nonneural lineages as cell sources, which are easy to obtain and expand, while causing minimal damage to the body. BMSCs, a type of pluripotent stem cell originating from the bone marrow, can be easily obtained and expanded. Although they originate from the mesoderm, they can differentiate into neurons. Until recently, inducing BMSCs to differentiate into ectodermal neural lineage cells was confronted with many problems that included complex induction approaches, low differentiation rates, poor functionality and poor stability, etc. [24–29].

In this study, BMSCs were the cell source and were differentiated into neural lineage cells induced by bFGF-chitosan controlled release scaffolds. Furthermore, we synchronized BMSCs to the G0/G1 phase by serum starvation to improve the differentiation percentage of BMSCs into neural lineage cells. We also examined the expression levels of key molecules (FGFR1, ERK, and c-fos) in the bFGF signaling pathway and the molecular switch BTG2 (which controls the transformation from cell proliferation to differentiation) and found that all expression levels were significantly increased. All of these results provided information regarding the molecular mechanism associated with BMSC differentiation into neurons at a high percentage induced by bFGF-chitosan controlled release scaffolds. We use a single factor combined with chitosan to induce BMSC differentiation, avoiding the use of cocktail growth factors [30]. In addition, our induction method allows BMSC to pass through neural stem cells, process from immature neurons to mature neurons, and differentiate into GAD67-positive inhibitory neurons.

Compared with synthetic polymers, natural polymers have improved tissue compatibility and biological degradability. Chitosan, a type of chitin derivative, is obtained via local deacetylation and is the only positively charged cationic polymer found in nature. These advantages make chitosan a critical material for tissue engineering research [31]. bFGF is a type of cellular factor with broad applications. Under normal physiological conditions, bFGF can facilitate cell proliferation and division and is thus involved in neuronal generation and tissue repair for brain and spinal cord injuries [32–34]. The combination of bFGF and the FGFR1 receptor may activate several signaling cascades, including phosphoinositide 3-kinase, phospholipase, and MAPK signaling pathways [35]. Under physiological conditions, however, bFGF has a short half-life (only 17 hours) [36] and can hardly pass through the blood-brain barrier, which results in quite low efficacy in a clinical setting. It has been reported that soluble bFGF could induce mesenchymal stem cells to differentiate into NF^+^ neural cells with electrophysiological characteristics in vitro, but bFGF had to be administered throughout the entire process [2], which was very expensive. The ATR-FTIR results suggest that bFGF might combine with chitosan via hydrogen bonding. Furthermore, we conducted thermogravimetric analysis and verified the alteration in the thermal stability of the bFGF-chitosan controlled release scaffold, which indicated that bFGF likely interacted with the chitosan scaffold. These results theoretically explain how the bFGF-chitosan controlled release scaffold could function continuously under physiological conditions.

The MAPK signaling pathway, one of the most fundamental signaling pathways in vivo, plays a significant role in cell growth, division, proliferation, and differentiation as well as in various physiological and pathological processes. It can be activated by a series of extracellular signals or stimulating factors [37]. The ERK, JNK/SAPK, P38MAPK, and ERK5/BMK1 signaling pathways are four prominent members of the MAPK family. They are regulated by extracellular signals and activated by different stimulating factors to form different transduction pathways, which subsequently activate different transcription factors to achieve different biological effects. bFGF functions mainly through the MAPK/ERK pathway. We examined the expression levels of c-fos and ERK in the signaling pathway and found that the slow and long-term release of bFGF by the bFGF-chitosan scaffold continuously stimulated the MAPK signaling pathway, thus leading to a substantial increase in their expression levels and consequently improving the differentiation percentage into the neural lineage.

The cell cycle is closely related to cell proliferation and differentiation. When cells enter the G1 phase and smoothly pass the checkpoint to proceed to subsequent phases, they proliferate. When cells are stimulated by certain factors and enter the G0 phase, they begin to differentiate [38].

BTG2/Tis21, a transcription factor belonging to the BTG family, regulates the development of different cell types, including neural precursors [39]. It functions as a molecular switch for the transformation from cell proliferation to differentiation by controlling the exit of a cell cycle as well as the subsequent terminal differentiation [40]. According to a previous report, BTG2 has a vital role in the maturation of stem/precursor cells in the adult dentate gyrus and SVZ, but it primarily regulates cell migration instead of proliferation or differentiation in cerebellar precursor cells [41]. We examined the expression level of BTG2 during the differentiation of BMSCs into neural cells. The results suggest that BTG2 also played a significant role in the transformation from cell proliferation to differentiation, and the increased differentiation percentage into neural lineage cells was accompanied by an increasing BTG2 expression level. We speculate that the bFGF-chitosan controlled release scaffold activated the BTG2 molecular switch and thus caused more cells to transform from proliferation to differentiation, ultimately resulting in an improved differentiation percentage into neural lineage cells. Moreover, when cells were synchronized to the G0/G1 phase, the expression level of BTG2 also increased. As a result, more cells exited their respective cell cycles and completed terminal differentiation under bFGF stimulation, further improving the differentiation percentage of BMSCs into neural cells.

By combining the bFGF-chitosan controlled release scaffold with cell cycle synchronization, we induced BMSCs to differentiate into neurons at a high percentage and tentatively clarified the underlying molecular mechanism. This study may provide insight regarding the clinical application of BMSCs in the treatment of CNS injury and neurodegenerative diseases. Because the evaluation of electrophysiological functions is crucial when studying neurons, we will explore the electrophysiological functions of BMSC-originating neurons in future research. In the next study, we will study whether these cells induced by bFGF-CCRS are effective neural circuits in the injured area as replacement neurons or cells that act as a delivery vehicle of neurotrophic factors only.

## 5. Conclusions

We proposed an approach to combine bioactive sustained-release materials with engineered cells that do not involve gene modulation and can dramatically improve the differentiation of BMSCs into neurons. The possible molecular mechanism associated with changing the cell fate to cause more cells to transform from proliferation to differentiation, thereby enhancing the possibility of neural differentiation, and upregulating the MAPK signaling pathway were the specific approaches implemented for cell differentiation.

## Figures and Tables

**Table 1:** Antibodies and dilutions.

Antibody	Antibody species	Dilution ratio
Nestin	Mouse	1:300
Tuj-1	Rabbit	1:300
Chat	Rabbit	1:200
GAD67	Mouse	1:100
CD34	Mouse	1:200
CD31	Rabbit	1:300

**Table 2:** Primers used for the QRT-PCR analysis.

Name	Forward primer	Reverse primer
Nestin	GAGGACCAGGAGGCATGTAG	TCTTCTGGAGACCTCAGGGA
Tuj-1	TGAGGCCTCCTCTCACAAGT	GGCCTGAATAGGTGTCCAAA
MAP2	ACCACAGCAACAAGTGGTGA	AAGGTCTTGGGAGGGAAGAA
ChAT	CTGGGATCCAGAGACTGTCG	AGGTAGCCACAATGGCAAAG
GAD67	GTTTGATCCGATCCAGGAGA	TCTATGCCGCTGAGTTTGTG
c-fos	AGAATCCGAAGGGAAAGGAA	GTTGATCTGTCTCCGCTTGG
ERK	TCCAAAGCTCTTGACCTGCT	TGTTCCAGGTAAGGGTGAGC
BTG2	CTTTGCTCTGTGCTGCTT	CTGGGTTTCTCATAGGTTAC
FGFR1	CTCTGTGGTGCCTTCTGACA	TTCACCTCGATGTGCTTCAG

**Table 3:** Cell percentage at each cell cycle phase under different conditions.

G0/G1+S+G2/M					
	12 h	24 h	36 h	48 h	72 h
5%	77.30+12.53+10.17	79.40+11.68+8.93	87.54+6.19+6.27	88.51+6.02+5.47	91.17+4.16+4.67
1%	90.66++4.98+4.36	90.44+5.53+4.03	92.88+4.04+3.09	95.80+2.4+1.80	92.8+2.87+4.35
0.5	82.712+10.6+6.68	89.18+5.2+5.69	92.1+3.53+4.38	91.15+4.41+4.44	92.1+3.53+4.38
0.1	83.56+7.57+8.85	86.3+6.1+7.6	86.21+6.61+7.71	87.29+6.77+5.94	89.51+6.4+4.09
0%	84.6+7.81+7.59	85.19+8.95+5.86	85.49+7.87+6.64	85.5+8.05+6.45	86.15+5.70+8.15

FBS of different concentrations were processed for different times, repeated for 5 times for each group. Under the condition of 1% FBS for 48 h, the G0/G1 cell number reached the peak.

## Data Availability

The data used to support the findings of this study are available from the corresponding author upon reasonable request.
